# The effect of alcoholics anonymous group participation on flourishing in Turkey: the mediating role of hope and social support

**DOI:** 10.1186/s12888-025-07667-6

**Published:** 2025-11-28

**Authors:** Samet Can Demirci, Burak Erman Menkü, Naz Aksoy, Ahmet Özaslan, Zehra Arıkan

**Affiliations:** 1 Department of Child and Adolescent Psychiatry, Faculty of Medicine, Lokman Hekim University, Söğütözü Neighborhood, 2179 Street No: 6, Ankara, Çankaya 06510 Turkey; 2 Department of Psychiatry, Faculty of Medicine, Lokman Hekim University, Ankara, Turkey; 3 Özgür Köy Treatment and Rehabilitation Center, Faculty of Medicine, Lokman Hekim University, Ankara, Turkey; 4 Department of Child and Adolescent Psychiatry, Faculty of Medicine, Gazi University, Ankara, Turkey; 5 Child Protection Research and Application Center, Gazi University, Ankara, Turkey; 6 Psychology Research Centre, Khazar University, Baku, Azerbaijan

**Keywords:** Alcohol use disorder, Alcoholics anonymous, Flourishing, Turkey, Hope

## Abstract

**Background:**

Alcohol use disorder (AUD) is a significant public health issue that negatively impacts individuals’ flourishing, social relationships, and life satisfaction. In recent years, the concept of recovery has been approached as encompassing not only the reduction or cessation of alcohol use but also the strengthening of positive psychology-based resources such as hope, social support, and developmental flourishing. Alcoholics Anonymous (AA) groups are an important community-based intervention that can contribute to the development of these resources through mutual experience sharing and the 12-step program. This study aims to examine the effect of AA participation on flourishing and the mediating role of hope and perceived social support in this relationship.

**Methods:**

The study, conducted using a cross-sectional comparative design, included 245 participants from all official AA centers operating in Turkey’s seven geographical regions (low participation *n* = 109; high participation *n* = 136). Participants completed validated self-report scales: Flourishing Scale, Continuous Hope Scale, Multidimensional Perceived Social Support Scale, Connor-Davidson Resilience Scale, and DASS-21. Age, depression and stress were controlled as covariates in the analyses. Data were evaluated using MANCOVA and mediation analyses.

**Results:**

High levels of AA participation were found to be significantly associated with higher flourishing, hope, and perceived social support; and lower levels of depression and stress. Mediation analyses indicated that hope (indirect effect = 0.75, 95% CI [0.23, 1.29]) and perceived social support (indirect effect = 0.59, 95% CI [0.21, 1.07]) mediated the relationship between AA participation and flourishing. The final model explained 28% of the variance in flourishing (R² = 0.28, *p* < 0.001).

**Conclusions:**

Regular participation in AA is associated with reducing or ceasing alcohol use and with enhanced flourishing by strengthening hope and social support. These results highlight the critical role of positive psychology-based resources in the recovery process from addiction. Furthermore, as the study was conducted using data obtained from all official AA centers in Turkey, it reflects the socio-cultural diversity across the country and offers a unique contribution to the literature.

**Clinical trial number:**

Not applicable.

**Trial registration:**

Not applicable.

## Background

Alcohol use disorder (AUD) is a chronic public health problem that profoundly affects not only physical health but also mental well-being, social relationships and life satisfaction [1, 2]. In the treatment of AUD, goals include reducing or stopping alcohol use and strengthening the individual on psychological, social, and emotional levels. In this context, mutual aid-based groups, especially Alcoholics Anonymous (AA), have become one of the most widely used support systems in the world [3–5].

AA groups aim to help individuals maintain their sobriety through a structured 12-step approach, mutual experience sharing and a sense of belonging [6]. Therefore, most of the studies to date have focused on the effects of AA participation on the duration of sobriety, relapse rates, and the ability to remain abstinent from alcohol [7–9]. This literature strongly suggests that regular participation in AA is effective in reducing individuals’ alcohol use.

However, in recent years, the concept of recovery from addiction has begun to be addressed with a more holistic approach that is not limited to quitting alcohol use. The current understanding of recovery includes not only the maintenance of the individual’s state of “sobriety”, but also positive psychological gains such as increased sense of hope, strengthened perception of social support, and ultimately the ability to realize one’s own potential [10–14]. Therefore, addressing recovery only from a narrow perspective such as ‘did he/she relapse’ may lead to the risk of ignoring the emotional and social needs of the individual.

Flourishing, as used in this study, is a multidimensional developmental state that integrates emotional, psychological, and social functioning. Flourishing encompasses not only the absence of psychological disorders but also many positive dimensions such as life satisfaction, positive affect, sense of meaning, interpersonal functioning and individual development [15–17]. Following Keyes, we treat flourishing as the high end of the positive mental health continuum, integrating emotional, psychological, and social functioning rather than mere symptom absence [17]. In line with Seligman, flourishing entails durable meaning and purpose, active engagement and accomplishment, and high-quality relationships [15]. Therefore, individuals struggling with addiction should be assessed not only whether they remain sober but also how meaningful and satisfying they find their lives [16]. For this reason, flourishing was adopted as a central outcome in this study.

Psychological resilience is defined as an individual’s capacity to adapt, maintain emotional balance, and continue functioning in the face of difficult life events, trauma, or chronic stress [18, 19]. In the process of overcoming addiction, psychological resilience is considered a fundamental personal resource that enables openness to change, restructuring, and the pursuit of sustainable flourishing in life [20, 21]. In this regard, psychological resilience is recognized as an important factor that can psychologically support the recovery process [19, 21].

Hope is a cognitive belief system that an individual can both find ways to reach a desired future and has the capacity to use these ways [22]. According to positive psychology theories, individuals with high levels of hope have more positive expectations for the future, their coping strategies are more effective and their perception that their lives are meaningful is stronger [23]. In situations that challenge the basic functions of life such as addiction, hope is one of the basic dynamics of restructuring and recovery [12, 13]. All of these characteristics are directly related to the basic components of flourishing [24]. Indeed, studies have shown that high levels of hope have positive effects on flourishing [25, 26]. In addition, hope has been defined as an important determinant of positive psychological changes that develop especially after traumatic life events [27–29].

For these reasons, in our study, hope was considered as a key psychological mediator in the relationship between AA participation and flourishing. Factors such as the collective support that AA meetings offer to individuals, encountering recovered role models and the opportunity to express themselves may increase the level of hope. This increase is expected to contribute to flourishing indicators such as life satisfaction, personal meaning and development.

Perceived social support is an individual’s subjective assessment that they can receive emotional, cognitive or practical support from their environment when needed [30]. For individuals struggling with addiction, social support supports many positive processes such as reduced feelings of loneliness, coping with stigmatization and treatment adherence [11]. Social support is also a strong variable directly related to flourishing. Especially interpersonal relationships and social acceptance are one of the main components of flourishing [16]. Studies have shown that high perceived social support decreases both psychological symptoms (e.g. depression and anxiety) and increases life satisfaction [31–33]. The basic dynamics of AA groups are mutual support, experience sharing and unconditional acceptance [6]. With these features, AA participation may increase individuals’ perceptions of social support. Moreover, social support plays an important role not only in times of crisis but also in creating positive expectations for the future and feeling valuable [31, 33, 34]. Therefore, in our study, perceived social support was considered as the second main mediator variable that could explain the relationship between AA participation and flourishing.

In addition, individuals struggling with addiction often experience high levels of psychological distress, particularly depression, anxiety, and stress [35]. This situation can negatively affect not only the individual’s compliance with the treatment process but also the development of positive psychological resources such as hope, perceived social support, and life satisfaction [36]. Indeed, research shows that individuals with high levels of depression and anxiety have reduced motivation to recover [37], while participation in community-based support systems has been shown to alleviate these symptoms [38]. Structured social support environments such as AA can reduce individuals’ feelings of loneliness and helplessness, making it easier for them to cope with such negative emotions [6, 39]. Therefore, in our study, we aimed to conduct an independent assessment of the effects on flourishing by controlling for depression, anxiety, and stress levels.

### Present study

The aim of this study is to examine the relationship between regular attendance at AA meetings and individuals’ flourishing levels, as well as the mediating role of hope and perceived social support in this relationship. Age, depression, anxiety, and stress were controlled for in the model.

**Fig. 1:**
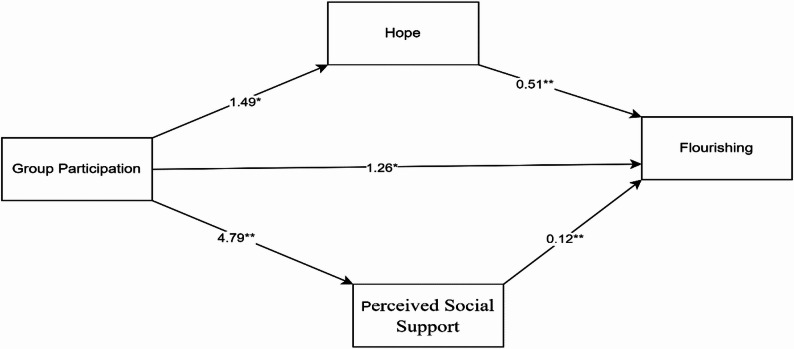
Conceptual model of AA participation and flourishing via hope and social support

Accordingly, the following hypotheses were tested, and the conceptual framework of the model is presented in Fig. 1.

1. Individuals who regularly participate in AA will report higher levels of flourishing, hope and perceived social support compared to individuals with low participation.
2. The relationship between AA participation and flourishing will be explained indirectly through the level of hope.
3. The relationship between AA participation and flourishing will be indirectly explained through the level of perceived social support.
4. Hope and social support will contribute significantly in the model as psychological variables positively associated with flourishing.

## Methods

### Participants

This study was conducted using a cross-sectional comparative research design aimed at comparing the psychosocial characteristics of individuals participating in AA programs at different levels. Data collection was carried out between October 2024 and May 2025 through online communication with local representatives of a total of 32 AA centers operating in all seven geographical regions of Turkey. All official AA centers operating throughout Turkey were included in this study, with some of these centers located in large cities (e.g., Istanbul, Ankara, Izmir) and others in smaller or rural cities. This design provided broad coverage across regions and meetings; however, the sample should not be interpreted as statistically representative of Turkey’s AA membership or of the country’s socio-cultural and demographic structures.

In previous studies, participation in AA programs was generally measured by scoring meeting frequency, sponsor relationships, 12-step practices, and other program activities, and summary scores reflecting the level of participation were created from these scores [34, 40–42]. However, the AA model has a holistic structure that encourages members to participate in multiple activities. Therefore, methods that score participation numerically either exaggerate individuals’ actual participation levels or fail to sufficiently distinguish between experiential differences. In this context, it is thought that the aforementioned measurement approaches may be insufficient to fully reflect the qualitative dimensions of participation in AA. Additionally, global involvement scores can be directly influenced by psychosocial resources that we analyze as mediators (e.g., hope and perceived social support), introducing potential construct overlap and endogeneity. They may also misclassify individuals with only 4–5 months of irregular attendance who previously completed steps or briefly sponsored others yielding relatively high scores despite limited sustained exposure.

Therefore, in our study, instead of scoring participation, we chose to compare individuals who have recently joined AA with those who have been participating at a high level for a long time, clearly distinguishing between them based on clear and objective inclusion/exclusion criteria. It is anticipated that this approach may reveal the psychosocial effects of AA more directly and reliably.

Participants were divided into two groups according to their level of participation in the AA program:

Group 1 (Low Participation Group): Inclusion criteria: [1] Be over 18 years of age [2] Sobriety period of less than one week [3], Have not completed the 12-step program [4] Voluntarily agree to participate in the study.

Group 2 (High Participation Group): Inclusion criteria: [1] Be over 18 years of age [2], Have regularly attended AA meetings at least once a week for the past year [3], Have completed the 12-step program [4], Either being guided by a sponsor or serving as a sponsor [5] Be sober for at least one year [6], Voluntarily agree to participate in the study. Taken together, these duration- and practice-based criteria provide an objective, clinically interpretable indicator of cumulative AA exposure and were selected to ensure a clear analytic contrast between group.

Exclusion criteria for both groups were: [1] Presence of acute psychiatric illness (e.g., psychotic disorders) [2], Diagnosed cognitive impairment or dementia [3], Participation in another addiction support group [4] Refusal or inability to provide informed consent.

During the data collection process, a total of 267 individuals were reached through 32 different AA centers across Turkey. As a result of the controls made by the researchers, the data of 22 individuals who did not meet the inclusion and exclusion criteria or had missing data were excluded from the analysis.

### Measures

All instruments were administered in their validated Turkish forms. Unless otherwise specified, psychometric information refers to the published Turkish validation studies for each instrument.

#### Sociodemographic form

A structured form was used to assess basic demographics (age, gender, marital status, education, income) and duration of sobriety in days.

#### Flourishing scale (FS)

The FS consists of 8 items rated on a 7-point scale (1 = strongly disagree, 7 = strongly agree). Higher scores indicate higher flourishing. The Turkish version showed adequate psychometric properties [43, 44].

#### Dispositional hope scale (DHS)

The DHS includes 12 items rated on an 8-point scale (1 = absolutely false, 8 = absolutely true) with two dimensions: agency and pathways. Higher scores indicate greater hope. Total scores were used in this study. The Turkish version is reliable and valid [45, 46].

#### Connor-davidson resilience scale (CD-RISC)

Resilience was measured with the 25-item CD-RISC, scored 0–4, with higher scores indicating better resilience. The Turkish version showed high reliability [18, 47].

#### Multidimensional scale of perceived social support (MSPSS)

The MSPSS is a 12-item scale measuring perceived support from family, friends, and a significant other, scored from 1 to 7. Higher scores reflect higher perceived support. The Turkish version is valid and reliable [30, 48].

#### Depression anxiety stress scales − 21 items (DASS-21)

The DASS-21 consists of 21 items rated on a 4-point scale (0–3) assessing depression, anxiety, and stress in the past week. Higher scores indicate more severe symptoms. The Turkish version has adequate validity and reliability [49, 50].

### Procedure

The purpose of the study and data collection tools were explained to these representatives and the data collection process was coordinated through weekly online meetings for approximately six months.

Following the meetings, the representatives at the AA centers met face-to-face with the participants to fill out the questionnaires, which were then emailed to the research team each week before the online meetings. This structured process ensured regular and reliable data flow from the field.

#### Statistical analysis

Considering the multivariate statistical analyses planned in the study (e.g., MANCOVA and parallel mediation analyses), the minimum sample size was determined based on a medium effect size (f² = 0.15), 95% confidence level and 80% statistical power (computed with G*Power 3.1) [51]. Accordingly, it was predicted that at least 200 participants should be included, and the target sample size was increased by 20% to 240, taking into account possible data losses and invalid forms. Descriptive statistics were calculated for all study variables. A MANCOVA was performed to examine the effect of group participation (low vs. high) on flourishing, resilience, perceived social support, hope, depression, anxiety, and stress, controlling for age. Pillai’s trace was used as the multivariate test statistic, and follow-up univariate analyses with Bonferroni correction were conducted. Mediation analyses were then carried out with the PROCESS macro (Model 4) to test whether hope and perceived social support mediated the effect of group participation on flourishing, controlling for age, stress, and depression. Anxiety was measured but was not included as an adjustment covariate to limit multicollinearity among the DASS-21 subscales and to avoid over-adjustment. Indirect effects were evaluated using 5,000 bootstrap samples with 95% bias-corrected confidence intervals. All analyses were conducted using SPSS version 27 for Windows, with significance set at *p* < 0.05.

### Use of generative AI

A large language model (ChatGPT, OpenAI) was used to assist with English language polishing and reference formatting. The authors verified the accuracy and integrity of the content, take full responsibility for the work, and confirm that no AI system met authorship criteria or had access to non-public data.

## Results

The remaining 245 participants were included in the study, which indicates that 91.8% valid responses were obtained. Of the participants, 109 were in the first group (new participation) and 136 were in the second group (high participation). The majority of the sample were male (*n* = 178, 72.7%), while 27.3% were female (*n* = 67). The sociodemographic characteristics of the participants are presented in Table 1.

**Table 1:** Sociodemographic characteristics of the participants

		*N*	%
Gender	Male	178	72.7
	Female	67	27.3
Group	Low participation	109	44.5
	High participation	136	55.5
Marital Status	Single	108	44.1
	Divorced	114	46.5
	Married	23	9.4
Education Level	Primary school	15	6.1
	Middle school	20	8.2
	High school	116	47.3
	University	70	28.6
	Postgraduate	24	9.8
Socioeconomic Level	Below minimum wage	30	12.2
	Minimum wage	137	55.9
	Twice the minimum wage	50	20.4
	Three times the minimum wage or more	28	11.4
Total		245	100.0

**Table 2:** Effect of group participation on Flourishing, Resilience, perceived social Support, Hope, Depression, Anxiety, and stress (MANCOVA univariate Results)

Independent variable	Dependent variable	Univariate F	df	*p*	Partial η²
Group Participation (Low vs. High)	Flourishing	12.37	1,242	0.001*	0.049
	Resilience	2.58	1,242	0.110	0.011
	Perceived Social Support	12.63	1,242	< 0.001*	0.050
	Hope	8.98	1,242	0.003*	0.036
	Depression	5.96	1,242	0.015*	0.024
	Anxiety	3.07	1,242	0.081	0.013
	Stress	11.72	1,242	0.001*	0.046

Notes: Values represent univariate F tests from MANCOVA, controlling for age as a covariate

* Statistically significant at *p* < 0.05. Group participation coded as 0 = low, 1 = high

### One-way multivariate analysis of covariance (MANCOVA)

MANCOVA was applied to examine the effect of group participation (0 = low, 1 = high) on flourishing, resilience, perceived social support, hope, depression, anxiety, and stress, controlling for age. Box’s test indicated a violation of the assumption of equality of covariance matrices (*p* < 0.001), but Levene’s tests for homogeneity of error variances were non-significant for all dependent variables (all *p* > 0.05), indicating that the assumption of equal variances was met. The overall multivariate test was significant (Pillai’s trace = 0.152, F(7, 236) = 6.03, *p* < 0.001, partial η² = 0.15). These results indicate that group participation had a significant multiple effect on the dependent variables (Table 2).

Subsequent univariate follow-up tests revealed significant differences between groups on the variables of flourishing (F(1,242) = 12.37, *p* = 0.001), perceived social support(F(1,242) = 12.63, *p* < 0.001), hope (F(1,242) = 8.98, *p* = 0.003), depression (F(1,242) = 5.96, *p* = 0.015) and stress (F(1,242) = 11.72, *p* = 0.001). Pairwise comparisons with Bonferroni correction showed that participants with high group participation reported significantly higher flourishing (M = 32.39, SE = 0.41), perceived social support, and hope, as well as lower levels of depression and stress compared to the low participation group (M = 29.98, SE = 0.47 for the low group).

### Mediation analysis

The results of the mediation analysis including age, stress and depression as covariates are summarised in Table 3 and shown in Fig. 1. In the initial model including only the independent variable (group participation) and covariates (age, stress, and depression), the model explained 8.7% of the variance in flourishing (R² = 0.087, F(4,240) = 5.73, *p* = 0.001). Group participation was a significant and positive predictor for both hope (B = 1.49, *p* = 0.007) and perceived social support (B = 4.80, *p* < 0.001), explaining 5% and 8% of the variance in these mediators, respectively. In the final model, group participation (B = 1.30, *p* = 0.049), hope (B = 0.51, *p* < 0.001), and perceived social support (B = 0.12, *p* < 0.001) independently predicted flourishing significantly after controlling for all covariates. Indirect effects were judged significant because the 95% bias-corrected bootstrap confidence intervals excluded zero (Table 3), and the direct association between AA participation and flourishing remained significant after accounting for the mediators. The inclusion of hope and perceived social support as mediators increased the proportion of variance in flourishing explained by the overall model from 8.7% to 27.9% (ΔR² = 0.19).

**Table 3:** Unstandardized coefficients for the mediation model

	Consequent				Consequent			Consequent				
	M (Hope)				M (Perceived Social Support)			Y (Flourishing)				
Antecedent	Coeff.	*SE*	*t*	*p*	Coeff.	*SE*	*t*	*p*	Coeff.	*SE*	*t*	*p*
*Age*	-0.008	0.030	-0.27	0.786	–0.087	0.071	–1.24	0.218	0.016	0.034	0.48	0.633
*Stress*	–0.121	0.133	–0.91	0.366	0.666	0.318	2.09	0.037	0.070	0.154	0.45	0.653
*Depression*	0.047	0.116	0.41	0.683	–0.592	0.276	–2.14	0.033	0.143	0.134	1.07	0.286
*Group Participation*	1.491	0.547	2.72	0.007	4.797	1.306	3.67	< 0.001	1.296	0.654	1.98	0.049
Hope									0.506	0.074	6.84	< 0.001
Perceived Social Support									0.122	0.031	3.94	< 0.001
Constant	24.15	1.43	16.91	< 0.001								
Constant					34.12	3.41	10.01	< 0.001				
Constant									11.79	2.63	4.48	< 0.001
	*R²* =0.054 *F* = 3.44; *p* = 0.009				*R²* =0.078 *F* = 5.04; *p* < 0.001			*R²*^mediat^ = 0.279 *F* = 15.34; *p* < 0.001				

Note: Bootstrap samples = 5,000; bias-corrected 95% CIs reported. SE = standard error; Coeff = unstandardized coefficient; X = independent variable; M = mediator; Y = outcome variable. Indirect effects are considered significant when the 95% bias-corrected bootstrap CI does not include zero. The model including only the independent variable (group participation) and covariates explained 8.7% of the variance in flourishing (R² = 0.087). When hope and perceived social support were added as mediators, the explained variance increased to 27.9% (ΔR² = 0.19) (Table 4)

**Table 4:** Regression coefficients for direct and indirect links between group Participation, Hope, perceived social Support, and flourishing

Paths	Effect	SE	BootLLCI	BootULCI
Group Participation → Flourishing	1.30	0.65	0.01	2.58
Group → Hope → Flourishing	0.75	0.27	0.23	1.29
Group → Perceived Social Support → Flourishing	0.59	0.22	0.21	1.07
Total indirect effect	1.34	0.37	0.64	2.11
Total direct effect	2.64	0.70	1.25	4.02

LLCI = lower limit confidence interval, ULCI = upper limit confidence interval

Importantly, the indirect effect of group participation on flourishing was statistically significant through both hope (indirect effect = 0.75, 95% BootCI [0.22, 1.29]) and perceived social support (indirect effect = 0.59, 95% BootCI [0.21, 1.07]). In the final model, age, stress and depression were not found to be significant predictors of flourishing. These findings suggest that the relationship between group participation and flourishing is mediated by hope and perceived social support, independent of age, stress and depression.

## Discussion

Our study shows that participation in the AA program is significantly associated with flourishing, with significant indirect effects via hope and perceived social support. This finding reveals that AA contributes to a multidimensional recovery process that not only involves reducing or stopping alcohol use but also strengthens individuals’ psychosocial resources.

One of the main findings of this study is that the flourishing levels of individuals with high levels of participation in AA were found to be significantly higher than those with low levels of participation. This finding suggests that recovery is not limited to the cessation of alcohol use; it may also increase the individual’s capacity to restructure, make sense of and find satisfaction in life [15, 17]. We used a concise 8-item instrument aligned with these theoretical domains, supported by Turkish validation, and selected to balance theoretical coverage with minimal respondent burden in a multi-instrument field design.

The AA model contains many key elements that can support flourishing. In particular, the 12-step program encourages the individual to confront the past, go through a process of forgiveness, experience spiritual growth and gain a sense of belonging [6, 7]. Sharing experiences within the group helps the individual to realize that he/she is not alone and to develop a sense of unconditional acceptance. In these ways, AA can support the processes of “finding meaning in life and self-actualization”, one of the components of flourishing [12].

Flourishing was found to be higher especially in the high participation group, suggesting that AA may be effective not only on behavioral level but also on subjective well-being. Depending on the level of participation, it can be assumed that positive psychological resources such as hope, social support and meaning are activated more. Participation in regular meetings may support personal growth by enabling individuals to restructure both their inner awareness and interpersonal relationships [3, 14]. Indeed, Wnuk (2022) showed that participation in AA not only reduces alcohol use but also increases existential meaning, hope, and subjective well-being in individuals [10]. In addition, the systematic review by Parker et al. (2018) reported that individuals with high flourishing responded better to treatment and had a lower risk of relapse [16]. In this context, the finding that the flourishing level was higher in the high participation group in our study shows a strong consistency with the findings in the literature.

Another important finding of our study shows that hope levels may play a mediating role in the relationship between AA participation and flourishing. This finding reveals that hope is not merely a positive expectation about the future, but rather a multi-layered psychological construct with cognitive and behavioral dimensions. Internal restructuring, rather than external imposition, may be the basis for the distinctive contribution of AA to this process, which is the cultivation of hope in individuals. Narratives of personal recovery, especially shared in meetings, can trigger the idea that “if someone else can do it, so can I”, allowing the individual to reshape their expectations for the future. By strengthening the “perception of achievability” component of hope, these experiences can support the development of a system of expectations with direction and goals [13, 23].

However, the 12-step process in AA can facilitate the individual not only to confront the past but also to create a structured roadmap for the future. The step-by-step progression model can increase the perception of achievability by dividing the recovery process into smaller, manageable goals. This structure can contribute to the development of the cognitive structure defined as “pathways” in hope theory [45].

Another finding of our study is that individuals with a high level of participation in AA have significantly higher levels of perceived social support and this variable plays a mediating role in the effect on flourishing. This result suggests that social support is not only a buffer that comes into play in times of crisis; it can be a permanent psychosocial resource that sustains the individual’s flourishing [30, 31].

Unlike traditional aid relationships, the way AA generates social support is horizontal and reciprocal. Participants not only receive support, but also become givers of support. This two-way interaction may allow the individual to move from being just a recipient of help to gaining a functional position within the community. Thus, social support can create an active experience of belonging rather than a passive space of protection [7, 11].

In particular, the sponsor system can meet basic social needs such as attachment, trust and regular follow-up by offering a personalized support experience. This structure supports the establishment of sustainable social bonds by preventing relationships from remaining superficial. Positive relationships and social participation, which are important components of Flourishing, can be revitalized through this systematic structure (16).

The impact of social support on flourishing is not only related to the existence of relationships, but also to the perception of these relationships by the individual as reliable, meaningful and accessible. In-group norms repeated in AA meetings, collective discourses such as “you are not alone” or “we understand you” can reshape this perception on an emotional as well as identity level. This can create a fundamental restructuring of the individual’s relationship with the social world [31, 52].

Ultimately, this finding suggests that AA not only lifts individuals from social isolation but also provides a framework that allows them to re-make sense of the social world [16, 30].

The model developed in our study explains approximately 28% of the variance in flourishing. This rate indicates a remarkable explanatory power in terms of social behavioral models for understanding flourishing, which is a subjective and multidimensional concept. One of the prominent aspects of the model is that even after controlling for psychopathological symptoms such as depression, anxiety and stress, and age, AA participation remained significantly associated with flourishing. This finding shows that the effect of positive psychological resources can be evaluated relatively independently of pathological processes; therefore, it supports the theoretical approaches that flourishing should be defined not only by the absence of disease but also by the presence of developmental capacity [15, 17].

In the light of these findings, it is important to approach the effects of AA not only in the individual but also in the cultural context. Our study makes an original contribution to the limited literature in this field by examining the relationship between regular participation in AA and positive psychological resources in a cultural context such as Turkey where religious norms and traditional values are determinant [53, 54]. Although social relationships are strong in Turkey, social attitudes toward alcohol use can put pressure on individuals and make it difficult for them to seek help, share experiences, and receive support [55]. At this point, AA’s core principles of voluntarism, anonymity, and unconditional acceptance can facilitate more active participation in recovery processes by creating a social environment where individuals feel safe [6, 56]. In conclusion, data obtained from all AA centers across Turkey suggest that an approach supporting individuals’ psychosocial recovery is feasible in Turkey, where social sensitivities regarding alcohol use are pronounced.

In summary, this study shows that participation in AA supports a multidimensional recovery process that strengthens the individual’s psychosocial resources, not only for the cessation of alcohol use. The strengthening of positive resources such as flourishing, hope and social support necessitates the adoption of more holistic goals in clinical interventions [7]. The voluntary and anonymity-based structure of AA supports individuals’ intrinsic motivation and facilitates them to overcome social inhibitions [7]. These findings suggest that culturally context-sensitive, positive psychology-oriented and group-based interventions can be effective in societies like Turkey [52, 53].

The findings of this study should be interpreted with some limitations in mind. First, due to the cross-sectional design, the relationships between the variables do not claim causality. Although the findings show that there are significant relationships between participation in AA and positive psychological resources, the temporal course or reciprocal effects of these relationships cannot be clearly determined. Secondly, the data used in the study is purely self-reported. Participants’ responses may have been influenced by social approval bias. Especially in the field of addiction, which carries the risk of stigmatization, the objectivity of statements may be limited. Third, the sample was limited to individuals who were actively involved in AA. Fourth, because recruitment sought broad coverage across multiple AA meetings without proportional stratification and no national sampling frame was available, statistical representativeness cannot be claimed. Accordingly, the findings should be interpreted as analytic associations observed in a multi-site AA sample rather than as region- or nation-level estimates. This makes it difficult to generalize the results to other recovery pathways or intervention models. Fifth, our operationalization of AA participation prioritized duration- and practice-based grouping over a global involvement score; while this ensured a clear analytic contrast, it may not capture finer day-to-day variability in engagement. Accordingly, future work in Türkiye may combine duration- and practice-based criteria with a validated AA involvement measure to capture both the breadth and depth of engagement while preserving an objective contrast in cumulative exposure. All these limitations suggest the need for future longitudinal, multi-method studies comparing different intervention groups.

## Conclusions

This study shows that, in the Turkish context, regular participation in an AA program is not only limited to the reduction or cessation of alcohol use, but also supports a multidimensional recovery process that strengthens the individual’s positive psychological resources such as hope, social support, and flourishing.The findings reveal that the voluntary and anonymous nature of AA increases individual motivation and reduces social inhibitions, thereby providing a sustainable flourishing. This study, conducted in a society with culturally traditional values, provides important evidence that positive psychology-based, group-oriented and culturally sensitive interventions can be effective in the treatment of alcohol addiction.

## Data Availability

The datasets generated and analyzed during the current study are not publicly available due to privacy restrictions but are available from the corresponding author on reasonable request.

## Abbreviations

AA: Alcoholics Anonymous
AUD: Alcohol Use Disorder
BootCI: Bias-corrected bootstrap confidence interval
CD-RISC: Connor–Davidson Resilience Scale
CI: Confidence interval
Coeff: Unstandardized coefficient
DASS-21: Depression Anxiety Stress Scales–21 items
DHS: Dispositional Hope Scale
FS: Flourishing Scale
LLCI: Lower-limit confidence interval (bootstrap)
M: Mediator (variable)
MANCOVA: Multivariate analysis of covariance
MSPSS: Multidimensional Scale of Perceived Social Support
partial η²: Partial eta-squared (effect size)
PROCESS: Hayes’ PROCESS macro for mediation/moderation
SE: Standard error
SPSS: IBM SPSS Statistics
ULCI: Upper-limit confidence interval (bootstrap)
X: Independent variable
Y: *p* < 0. (dependent) variable
